# Association of Age and Spherical Equivalent With Differences in Cycloplegic Refractive Effects Between Cyclopentolate and Atropine

**DOI:** 10.7759/cureus.115799

**Published:** 2026-09-04

**Authors:** Toru Kawanobe, Toshiaki Goseki, Shinya Takahashi, Shingo Noda, Yasuhito Ikeda, Eri Ishikawa, Yuichiro Tanaka, Tadahiko Kozawa

**Affiliations:** 1 Department of Ophthalmology, Kozawa Eye Hospital and Diabetes Center, Mito City, JPN; 2 Department of Ophthalmology, International University of Health and Welfare, Atami Hospital, Atami City, JPN; 3 Department of Ophthalmology, Kitasato University School of Medicine, Sagamihara City, JPN

**Keywords:** amblyopia, atropine sulfate, cyclopentolate hydrochloride, refractive value, strabismus

## Abstract

Purpose: Amblyopia and strabismus are common visual impairments linked to functional and social consequences in children. In this retrospective study, we compared the cycloplegic effects of two cycloplegic eye drops, cyclopentolate and atropine, and examined whether age and initial spherical equivalent (SE) influenced these effects.

Materials and methods: We retrospectively reviewed electronic medical records from Kozawa Eye Hospital and Diabetes Center between 2001 and 2021. The primary outcome was the difference in SE between atropine and cyclopentolate (atropine minus cyclopentolate). As both eyes from the same patient could be included, multivariable generalized estimating equations (GEE) with an exchangeable working correlation structure clustered by patient identifier were used. Additional analyses included the Wilcoxon signed-rank test, Bland-Altman analysis, and Yeo-Johnson transformation of the outcome before multivariable modeling.

Results: The analysis included 186 eyes from 93 patients (median age, 6.00 years). Median SE was 2.47 D under cyclopentolate and 2.75 D under atropine, with a significant paired difference (Wilcoxon signed-rank test, p < 0.001). Bland-Altman analysis showed a mean difference of +0.42 D, with 95% limits of agreement from -0.44 to +1.28 D, and no significant proportional bias (slope = -0.016, p = 0.300). In the multivariable GEE model, older age was associated with a smaller atropine-cyclopentolate difference (β = -0.034, 95% confidence interval (CI): -0.065 to -0.003, p = 0.031), and higher initial SE was also associated with a smaller difference (β = -0.032, 95% CI: -0.060 to -0.004, p = 0.023). Sex and eye laterality were not significantly associated with the difference.

Conclusion: Atropine produced a stronger refractive effect than cyclopentolate in this pediatric cohort. The atropine-cyclopentolate difference was smaller in older patients and in eyes with higher initial SE, supporting consideration of age and baseline refractive status when selecting cycloplegic agents in pediatric patients.

## Introduction

Amblyopia is defined as reduced visual acuity resulting from abnormal development of the visual cortex during infancy or childhood, and it is a common visual acuity disorder in children [1,2]. Treatment options include glasses, occlusion, and penalization. However, the optimal approach depends on the patient's age at diagnosis, the timing and type of amblyopia onset, and the achievable level of visual acuity [3]. Previous studies have reported a 1.36% global amblyopia prevalence in children [3]. In contrast, strabismus in children is linked to decreased functional visual acuity and eye-related quality of life, and it has a greater impact on social life and causes irritability and distress [4]. An estimated strabismus prevalence of 1.93% has been observed in children worldwide, but these statistics are heterogeneously influenced by age, region, and year of publication [5,6].

It is essential to perform refractive testing using cycloplegic eye drops to enable the accurate diagnosis and treatment of amblyopia and strabismus in children [5-7]. The main cycloplegic eye drops are atropine sulfate (atropine) and cyclopentolate hydrochloride (cyclopentolate), with atropine demonstrating a stronger cycloplegic effect [7,8]. For instance, Sani et al. investigated the difference in obtaining refractive values using cyclopentolate and atropine in 63 subjects with a mean age of 8.23 years and found higher refractive values for atropine, although no significant difference was observed [8]. Additionally, Fan et al. conducted a similar experiment in 25 participants with an average age of 5.7 years and suggested that atropine demonstrated a higher cycloplegic effect by 0.42 D [9]. However, there are several concerns associated with using atropine. First, administering the drops requires time and effort. In addition, the effects of atropine, such as cycloplegia and mydriasis, can last for up to 14 days, whereas cyclopentolate has a duration of action of 24 hours [10]. Furthermore, adverse reactions such as flushing and fever have been reported [11-15]. As mentioned above, differences have been found between the refractive values of cyclopentolate and atropine. This difference is greater in younger patients and smaller in older patients [16]. Furthermore, when refractive error is ≥5 D, myopia is measured as less severe and hyperopia as more severe with atropine [17]. However, to our knowledge, few studies have examined whether differences in the cycloplegic effects of cyclopentolate and atropine in patients with amblyopia or strabismus vary according to age or initial spherical equivalent (SE).

Therefore, this study aimed to examine whether the atropine-cyclopentolate difference in SE is associated with age and initial SE.

This article was previously posted to the Research Square preprint server on November 18, 2025 [18].

## Materials and methods

Study design and population

In this retrospective observational study, we reviewed the electronic medical records of patients aged 3-12 years who underwent cycloplegic refraction on two occasions at Kozawa Eye Hospital and Diabetes Center between January 2001 and December 2021. Among these patients, those who underwent cycloplegic refraction with cyclopentolate hydrochloride eye drops (cyclopentolate) at the first examination, followed by atropine sulfate eye drops (atropine) at the second examination, were eligible for the study. Both examinations were performed as part of routine clinical care. Patients with an interval of more than 30 days between the two examinations were excluded. All included patients were Japanese and had dark irises. The participant selection process is shown in Figure 1.

**Figure 1:**
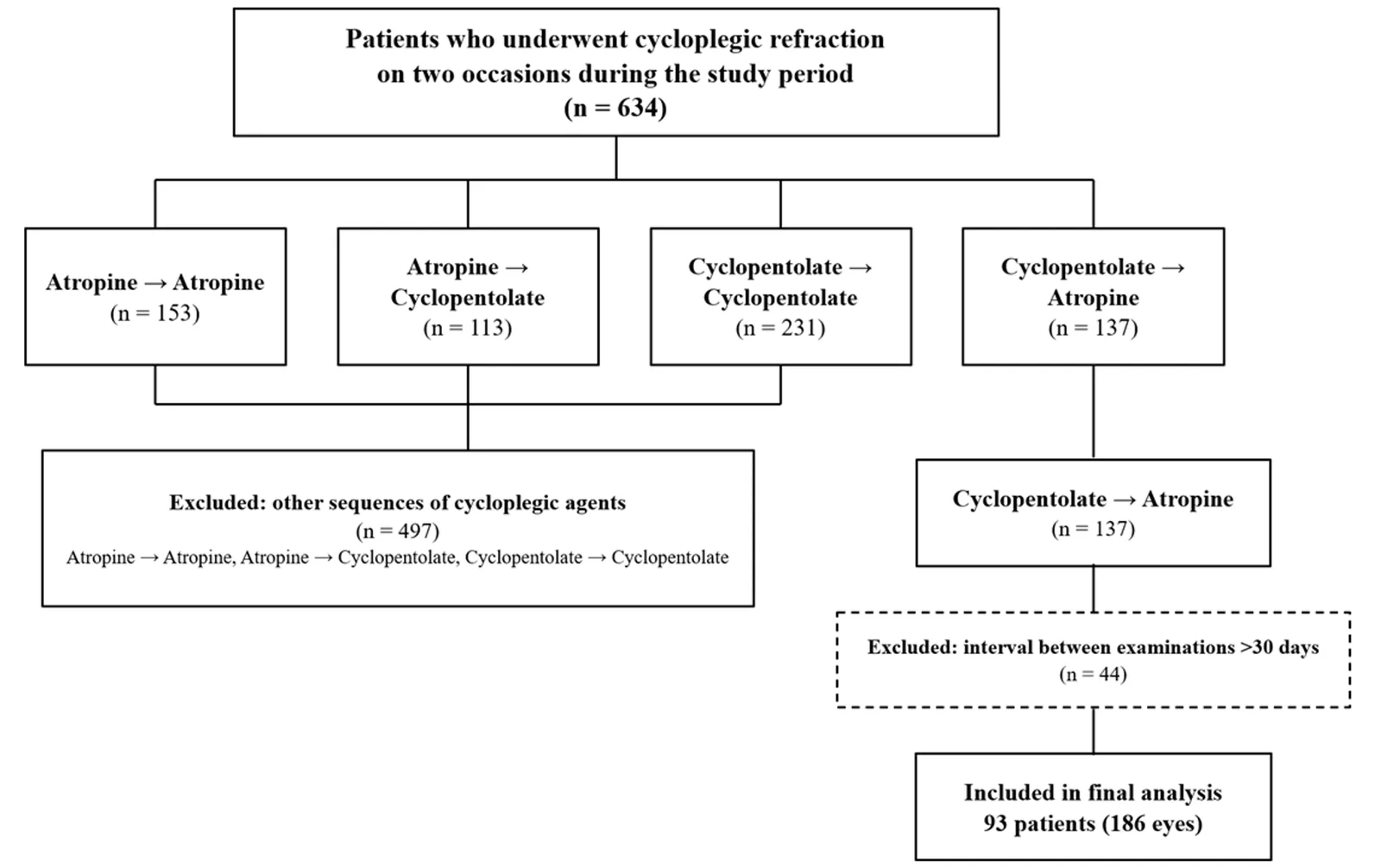
Flow diagram of participant selection. Among 634 patients who underwent cycloplegic refraction on two occasions during the study period, 137 underwent cyclopentolate, followed by atropine. After excluding 44 patients with an interval of more than 30 days between examinations, 93 patients (186 eyes) were included in the final analysis.

Collected variables

The outcome variable was the difference in SE between atropine and cyclopentolate (atropine minus cyclopentolate), hereafter referred to as the atropine-cyclopentolate difference. Cyclopentolate 1% was administered twice at five-minute intervals after hospital arrival, and refraction was measured 60 minutes later. Atropine was administered twice daily for seven days; children aged <6 years received 0.5% atropine, and those aged ≥6 years received 1.0% atropine. Refraction was measured using stationary autorefractometers (RC-5000, RT-7000, and MR-6000; Tomey, Aichi, Japan). Additional variables included sex, age at first examination, eye laterality, initial SE under cyclopentolate, and refractive shift (myopic or hyperopic).

Statistical analysis

Descriptive Statistics

Patient characteristics and refractive measurements were summarized using descriptive statistics. Continuous variables were assessed for normality using skewness. As all continuous variables included were non-normally distributed, they are presented as median (interquartile range, IQR). Categorical variables are presented as frequencies and percentages. The difference in SE between atropine and cyclopentolate (atropine minus cyclopentolate) was categorized into three groups: <0.5 D, ≥0.5 to <1.0 D, and ≥1.0 D.

Outcome Transformation

As the atropine-cyclopentolate difference was non-normally distributed, normality was assessed using the Shapiro-Wilk test, and a Yeo-Johnson transformation was applied before multivariable modeling (Figure 2). For clinical interpretation, back-transformed effects were calculated on the original diopter scale for key continuous predictors.

**Figure 2:**
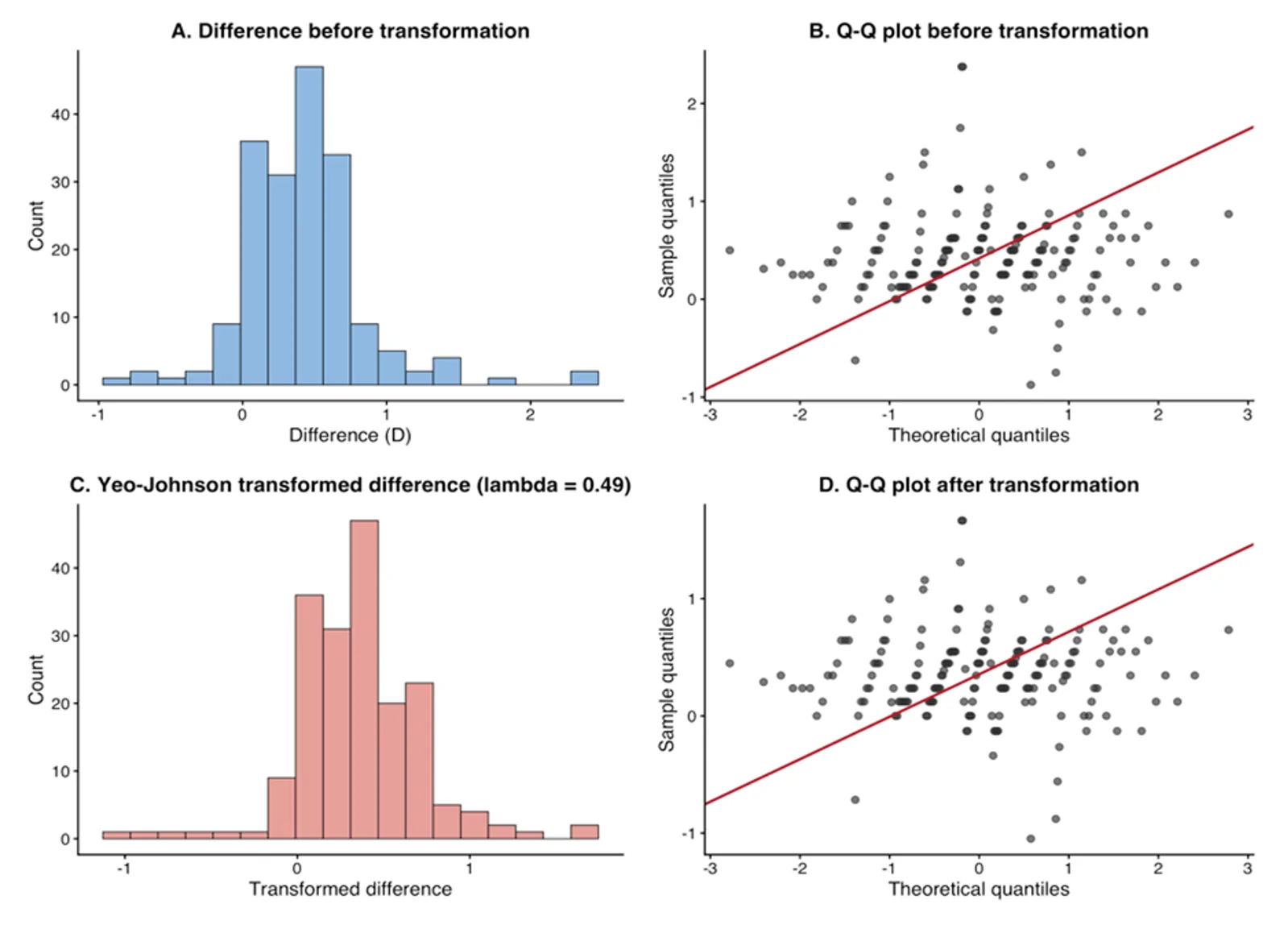
Distributional diagnostics of the atropine-cyclopentolate difference. A, B: Distribution and Q–Q plot before the Yeo–Johnson transformation. C, D: Distribution and Q–Q plot after the Yeo–Johnson transformation (lambda = 0.49), showing improved normality.

Generalized Estimating Equations (GEE) Model

To account for correlation between fellow eyes from the same patient, GEE with an exchangeable working correlation structure clustered by patient identifier was used. The dependent variable was the Yeo-Johnson-transformed atropine-cyclopentolate difference. Independent variables included age at first examination, initial SE under cyclopentolate, sex, and eye laterality.

As atropine concentration was determined according to age in our clinical protocol (<6 years: 0.5%; ≥6 years: 1.0%), it was not entered separately into the model because of collinearity with age. Accordingly, the coefficient for age should be interpreted with caution, as it may reflect age-related factors and differences in atropine concentration.

Regression Diagnostics

Model diagnostics were evaluated using plots of Pearson residuals versus fitted values, a normal Q-Q plot of standardized Pearson residuals, a scale-location plot, and cluster-level mean absolute Pearson residuals. In the cluster-level screening panel, the 95th percentile of the residual distribution was used as a visual threshold. To assess multicollinearity, variance inflation factors (VIFs) were calculated using a companion linear model with the same predictors.

Regression Model Diagnostics

Diagnostic plots are shown in Figure 3.

**Figure 3:**
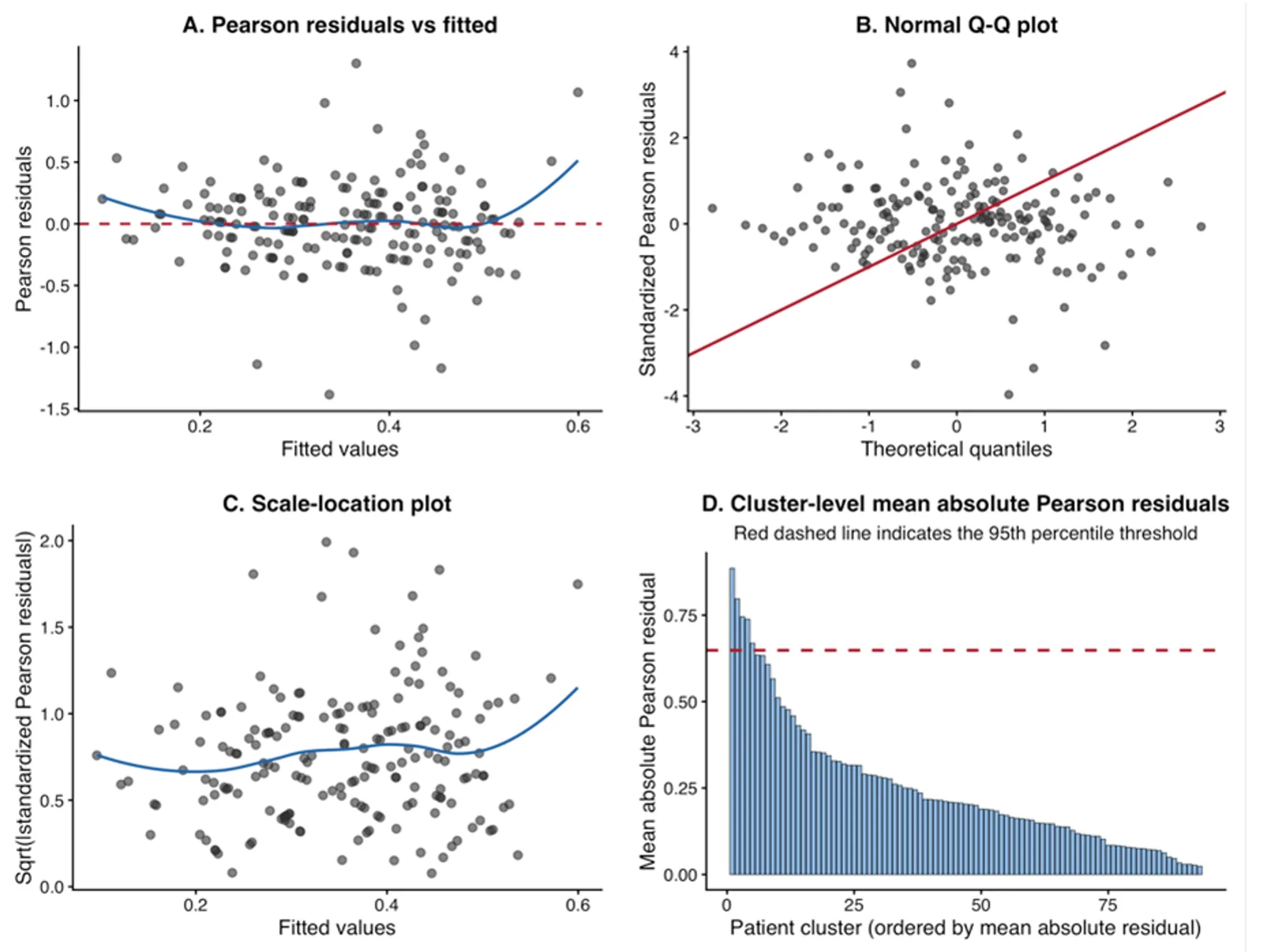
Diagnostic plots for the multivariable generalized estimating equations (GEE) model. A: Pearson residuals versus fitted values. B: Normal Q–Q plot of standardized Pearson residuals. C: Scale-location plot. D: Cluster-level mean absolute Pearson residuals. The red dashed line in panel D indicates the 95th percentile of the cluster-level residual distribution.

The Pearson residuals versus fitted values plot and the scale-location plot did not show a dominant systematic pattern, although mild curvature was observed at higher fitted values. The Q-Q plot showed some tail deviation from the reference line. Cluster-level mean absolute Pearson residuals were also examined, with the red dashed line indicating the 95th percentile threshold. Overall, these diagnostics did not suggest major violations that would invalidate model interpretation.

Bland-Altman Analysis

Bland-Altman analysis was performed to assess agreement between cyclopentolate and atropine measurements. The mean difference (bias) and 95% limits of agreement (mean difference ± 1.96 × standard deviation) were calculated, and proportional bias was assessed using linear regression of the difference on the mean of the two measurements.

Statistical Significance

All tests were two-tailed, and p < 0.05 was considered statistically significant. Analyses were performed in R version 4.3.2 (R Foundation for Statistical Computing, Vienna, Austria) using the geepack package for GEE modeling, the caret package for Yeo-Johnson transformation, and the car package for VIF calculations.

Ethics

The Institutional Review Board (IRB) of Kozawa Eye Hospital and Diabetes Center reviewed and approved the study protocol (approval number: KG2024-5). The IRB exempted the study population from obtaining individual consent, given the retrospective nature of this study. However, information about the nature of the study, intended use of data, and protection of privacy was included on the webpage, and it was clearly stated that participants could only choose to request exclusion from the study if they did not want their data to be utilized in the study. Data were anonymized and securely handled to maintain confidentiality, in accordance with the ethical standards of the Declaration of Helsinki (2024).

## Results

Study population characteristics

Table 1 summarizes the study population.

**Table 1:** Ocular characteristics of analyzed eyes stratified by difference in refractive values. Abbreviations: EP, esophoria; ET, esotropia; XP, exophoria; XT, exotropia. Data are presented as n (%) for categorical variables and median (Q1, Q3) for continuous variables. All continuous variables in this table were summarized as median (Q1, Q3) because they were classified as non-normal.

Variables	Overall (N = 186)	<0.5 D (N = 104)	≥0.5 and <1.0 D (N = 68)	≥1.0 D (N = 14)	Skewness
Gender					
Male	96 (51.6%)	50 (48.1%)	34 (50.0%)	12 (85.7%)	
Female	90 (48.4%)	54 (51.9%)	34 (50.0%)	2 (14.3%)	
Eye (Left/Right)					
Left	93 (50.0%)	60 (57.7%)	25 (36.8%)	8 (57.1%)	
Right	93 (50.0%)	44 (42.3%)	43 (63.2%)	6 (42.9%)	
Strabismus type					
ET	12 (6%)	7 (7%)	4 (6%)	1 (7%)	
XT	6 (3%)	3 (3%)	3 (4%)	0 (0%)	
EP	6 (3%)	4 (4%)	2 (3%)	0 (0%)	
XP	22 (12%)	11 (11%)	11 (16%)	0 (0%)	
Orthophoria	74 (40%)	40 (38%)	24 (35%)	10 (71%)	
Unknown	66 (35%)	39 (38%)	24 (35%)	3 (21%)	
Amblyopia type					
Refractive	116 (62%)	59 (57%)	49 (72%)	8 (57%)	
Anisometropic	60 (32%)	38 (37%)	16 (24%)	6 (43%)	
Strabismic	2 (1%)	2 (2%)	0 (0%)	0 (0%)	
None	8 (4%)	5 (5%)	3 (4%)	0 (0%)	
Age at first examination (years)	6.00 (4.50, 6.67)	6.42 (5.38, 7.34)	5.42 (4.29, 6.33)	4.50 (3.75, 6.00)	0.58
Interval between cyclopentolate and atropine examinations (days)	12.00 (7.00, 20.00)	13.00 (8.00, 22.00)	9.50 (7.00, 14.00)	13.00 (9.25, 26.25)	0.80
Best corrected visual acuity at first examination (logMAR)	0.15 (0.00, 0.40)	0.15 (0.00, 0.30)	0.15 (0.00, 0.40)	0.31 (0.06, 0.40)	0.78
Refractive value of cyclopentolate (D)	2.47 (1.50, 4.00)	2.63 (1.50, 4.13)	2.25 (1.44, 4.25)	1.31 (0.00, 3.50)	0.86
Refractive value of atropine (D)	2.75 (1.88, 4.63)	2.69 (1.75, 4.44)	2.88 (2.00, 4.75)	2.56 (1.75, 5.13)	0.83
Refractive shift					
Myopia	52 (28.0%)	44 (42.3%)	8 (11.8%)	0 (0.0%)	
Hyperopia	134 (72.0%)	60 (57.7%)	60 (88.2%)	14 (100.0%)	

The analysis included 186 eyes from 93 patients. Based on the atropine-cyclopentolate difference, 104, 68, and 14 eyes had differences of <0.5 D, ≥0.5 to <1.0 D, and ≥1.0 D, respectively. The interval between cyclopentolate and atropine examinations was a median of 12 days (minimum, seven days). The median age at first examination was 6.00 years. Median SE was 2.47 D under cyclopentolate and 2.75 D under atropine (Figure 4). Refractive shift was categorized as myopic in 52 eyes (28.0%) and hyperopic in 134 eyes (72.0%). The paired difference between atropine and cyclopentolate measurements was significant (Wilcoxon signed-rank test, p < 0.001).

**Figure 4:**
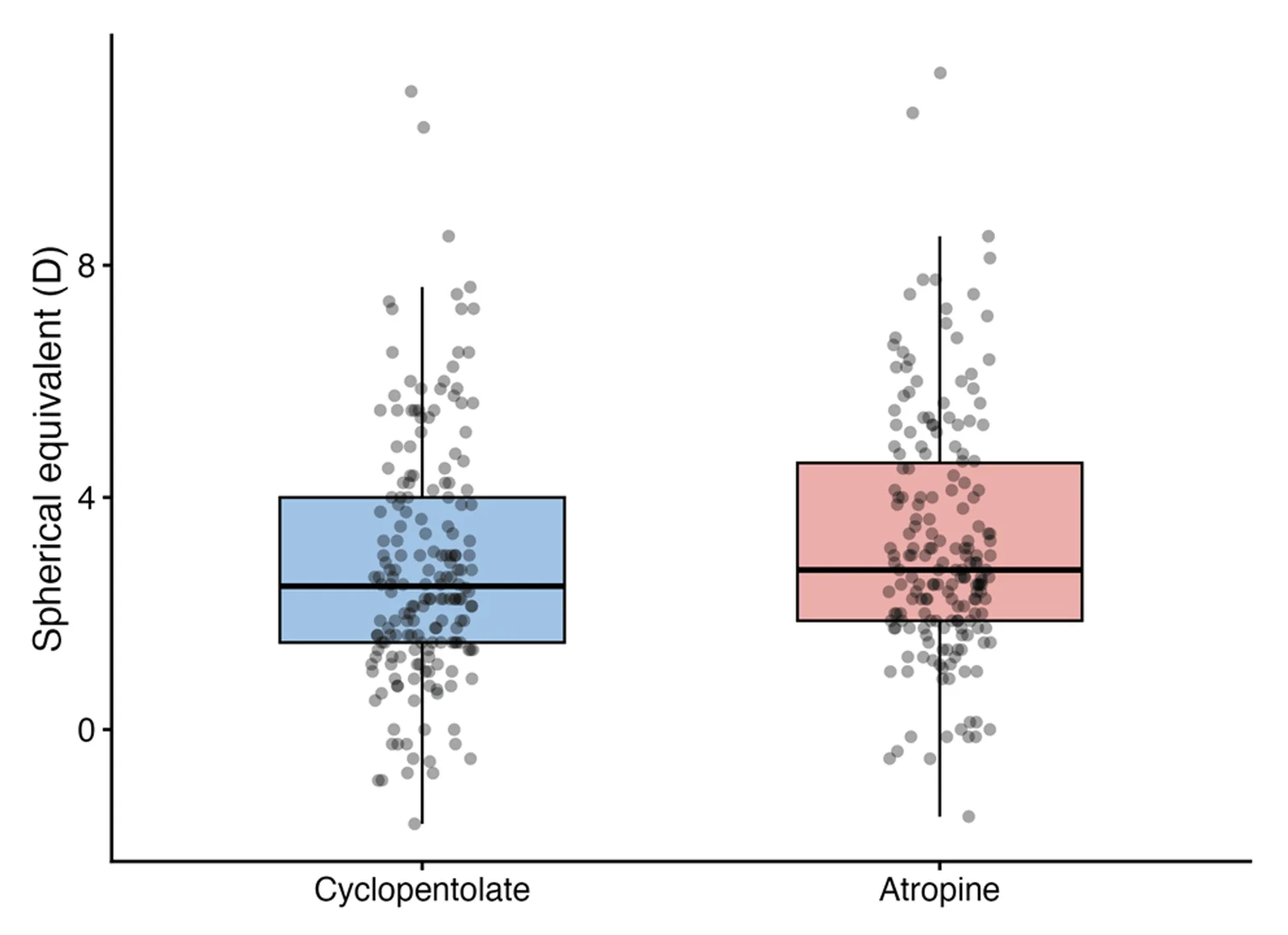
Distribution of spherical equivalent values measured under cyclopentolate and atropine. Boxplots show the median and interquartile range, with individual observations overlaid.

Factors associated with the atropine-cyclopentolate difference

Table 2 summarizes the multivariable GEE results.

**Table 2:** Integrated regression results with multivariable GEE clustered by patient ID. Abbreviations: CI, confidence interval; GEE, generalized estimating equations. Univariable analyses were performed using Spearman correlation for continuous predictors and Wilcoxon rank-sum tests for categorical predictors. Multivariable analysis was performed using generalized estimating equations with an exchangeable working correlation clustered by patient ID. The ID column was used as the patient identifier, and the same ID indicates fellow eyes from the same patient. The back-transformed effect is expressed on the original diopter scale.

Variable	Univariable association	P-value	Multivariable GEE β	95% CI	P-value	Back-transformed effect
Age at first examination (per year)	rho = -0.285	<0.001	-0.034	-0.065, -0.003	0.031	-0.041 D
Initial spherical equivalent (per D)	rho = -0.172	0.019	-0.032	-0.060, -0.004	0.023	-0.038 D
Gender (Female vs. Male)	-	0.266	-0.065	-0.180, 0.050	0.266	-
Eye (Right vs. Left)	-	0.169	0.042	-0.039, 0.123	0.309	-

Older age was associated with a smaller difference (β = -0.034, 95% confidence interval (CI): -0.065 to -0.003, p = 0.031), and higher initial SE was also associated with a smaller difference (β = -0.032, 95% CI: -0.060 to -0.004, p = 0.023). Sex (β = -0.065, 95% CI: -0.180 to 0.050, p = 0.266) and eye laterality (β = 0.042, 95% CI: -0.039 to 0.123, p = 0.309) were not significantly associated with the difference. Back-transformed effects suggested an approximate decrease of 0.041 D per year of age and 0.038 D per 1 D increase in initial SE.

Agreement between cycloplegic methods

Figure 5 shows the Bland-Altman plot comparing refractive values obtained with cyclopentolate and atropine. The mean difference (atropine minus cyclopentolate) was +0.42 D, indicating a systematic hyperopic shift with atropine. The 95% limits of agreement ranged from -0.44 D to +1.28 D. Regression of the difference on the mean refractive value showed no statistically significant proportional bias (slope = -0.016, p = 0.300).

**Figure 5:**
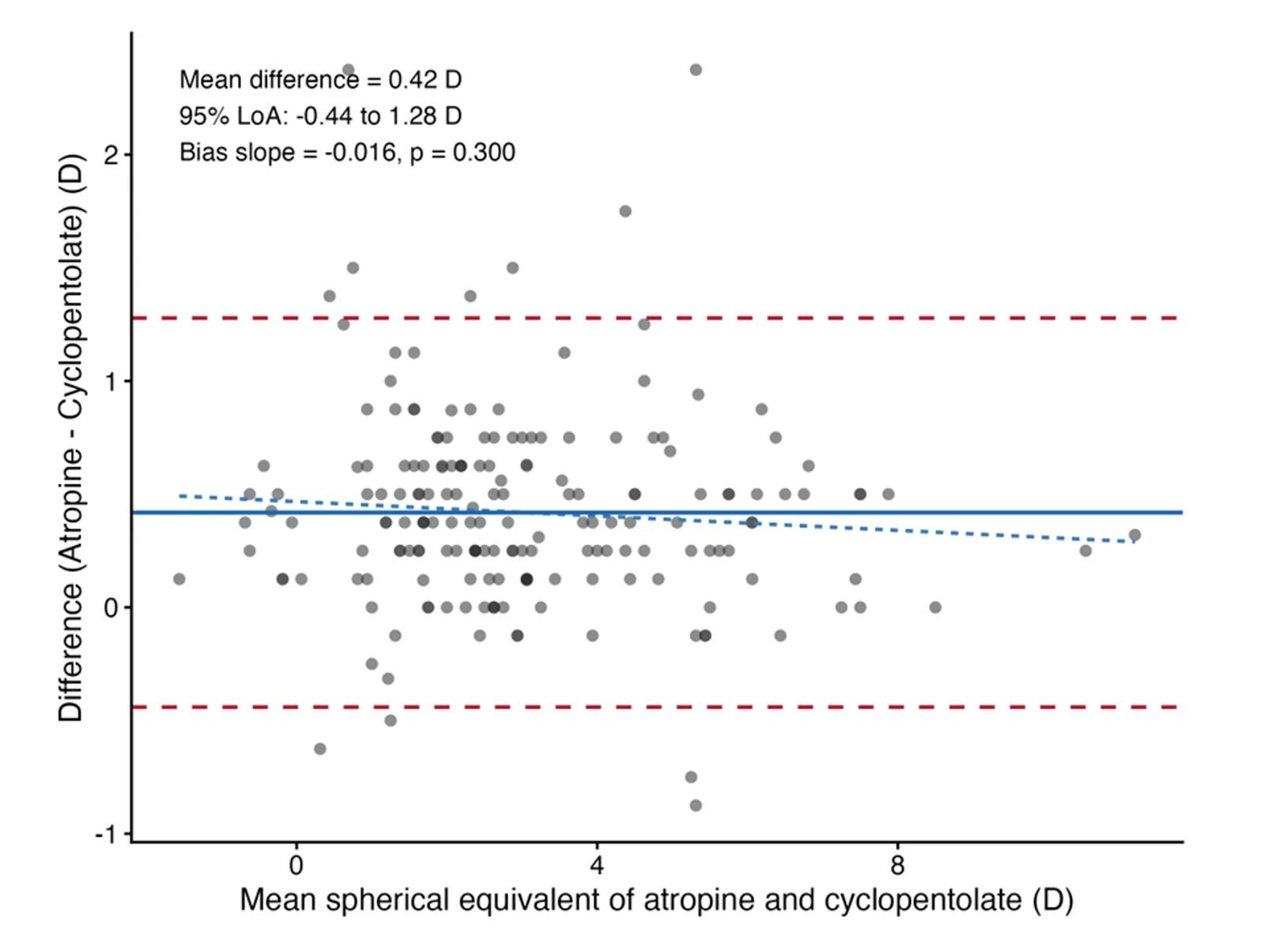
Bland-Altman plot comparing atropine and cyclopentolate measurements. The solid blue line indicates the mean difference, the red dashed lines indicate the 95% limits of agreement, and the blue dotted line indicates the fitted regression slope used to assess proportional bias.

## Discussion

Methodological considerations

A key strength of this study is the use of GEE to account for correlation between fellow eyes from the same patient. This approach allows inclusion of both eyes without assuming independence of paired observations and provides robust inference for the multivariable analysis. As atropine concentration was determined according to age in our clinical protocol, the estimated age effect should be interpreted with caution, as it may partly reflect age-related factors and the dosing protocol.

Main findings and comparison with previous studies

This study assessed whether differences existed in the cycloplegic effects of the cyclopentolate hydrochloride and atropine sulfate eye drops and whether these differences varied according to age and initial SE value. The cycloplegic effect of atropine was modestly stronger than that of cyclopentolate, and the difference was associated with age. The Bland-Altman analysis provided insight into agreement between cyclopentolate and atropine measurements. The mean difference of +0.42 D indicated a systematic bias toward more hyperopic values with atropine, consistent with its stronger cycloplegic effect. However, the 95% limits of agreement (-0.44 to +1.28 D) suggest that, at the individual level, differences between agents may vary substantially, which is clinically relevant when interpreting refraction. Our GEE analysis showed that initial SE was significantly associated with the atropine-cyclopentolate difference (β = -0.032, 95% CI: -0.060 to -0.004, p = 0.023), suggesting that this difference may be influenced not only by age but also by baseline refractive status. The finding that atropine has a stronger cycloplegic effect than cyclopentolate is consistent with previous reports showing differences of 0.2-0.41 D [8,9,19]. In this study, the difference was 0.42 D, indicating a comparable magnitude. Furthermore, when comparing groups aged ≤5 years and >5 years, Fan et al. reported differences of 0.55 D and 0.28 D, respectively, whereas Farhood reported 0.4 D and 0.2 D, indicating a stronger cycloplegic effect of atropine in the ≤5 years group [9,19]. Similarly, Wu et al. reported that the difference is greater in younger patients and smaller in older patients [16]. In this study, a negative correlation was also observed between the atropine-cyclopentolate difference and age. Although baseline SE and treatment regimens differed from those in previous studies, these findings suggest that the difference in cycloplegic effect between atropine and cyclopentolate may be smaller in older children.

Association with initial SE

Initial SE was associated with the difference in cycloplegic effects between the two agents (β = -0.032, 95% CI: -0.060 to -0.004, p = 0.023), suggesting that this difference varies not only with age but also with baseline refractive status. The negative association indicates that eyes with greater initial SE tended to show a smaller difference between agents. On the original diopter scale, the back-transformed effect corresponded to an approximate decrease of 0.038 D in the inter-agent difference for each 1 D increase in initial SE.

Clinical implications

These findings have several clinical implications. First, they may inform the selection of cycloplegic agents in pediatric ophthalmology. In the multivariable GEE analysis, older age and greater initial SE were associated with a smaller difference between cyclopentolate and atropine. Back-transformed estimates indicated that the difference decreased by 0.041 D per one-year increase in age and by 0.038 D per 1 D increase in initial SE. These results suggest that age and baseline refractive status should be considered when interpreting cycloplegic refraction and selecting an agent for individual patients. Clinically, the discrepancy between agents may be more pronounced in younger children and in those with lower initial hyperopia, whereas it may be smaller in older children and in those with higher initial hyperopia. Overall, these findings support a more individualized approach to cycloplegic agent selection. For example, atropine has been suggested for children with hyperopia >5.0 D and for patients with esotropia [9,17,20]. In contrast, cyclopentolate should be used with caution in patients with neurological disease because of reported adverse reactions, including speech disturbance, visual or tactile hallucinations, and ataxia [6,10].

Limitations

This study had certain limitations. First, there was potential selection bias in the use of cyclopentolate eye drops, as they were chosen as the first-line agent for a small number of patients with esotropia or high hyperopia. Second, the measurement instrument employed changed during the data collection period. Refractive measurements were obtained using stationary autorefractometers (RC-5000, RT-7000, and MR-6000; Tomey, Aichi, Japan). Although data from three instruments were combined, each patient was measured using the same instrument throughout the study. Previous studies have reported strong correlations between measurements obtained with different stationary autorefractometers [21,22]. In particular, Simsek et al. reported high correlation coefficients (r = 0.998 for spherical values and r = 0.964 for the cylindrical values) [22]. Therefore, inter-instrument measurement error is likely negligible. Another limitation is that both eyes were included to maximize statistical power in this relatively small cohort. Although GEE was used to account for within-patient inter-eye correlation, this bilateral-eye design may limit direct comparability with studies that analyze only one eye per patient. An additional limitation is that age and atropine concentration were inherently linked. As concentration was assigned according to age, the observed association between age and the atropine-cyclopentolate difference may partly reflect a dosing effect rather than an independent age-related effect. Therefore, the age-related findings should be interpreted with caution.

## Conclusions

Significant differences were observed between cyclopentolate and atropine refractive measurements, with atropine showing a stronger refractive effect. Older age and higher initial SE were associated with a smaller atropine-cyclopentolate difference. These findings support an individualized approach to cycloplegic agent selection in pediatric ophthalmology and justify further research to refine agent selection according to patient age and baseline refractive status.
